# Effect of Dietary Addition of *Lentinus edodes* on Rumen Flora, Lactation, and Health of Dairy Goats

**DOI:** 10.3390/ani15050676

**Published:** 2025-02-26

**Authors:** Huijun Shen, Mengyu Wang, Yong Ning, Yiqi Zhao, Baiji Danzeng, Kaixin Li, Huaiping Shi, Weijuan Li

**Affiliations:** 1 College of Animal Science and Technology, Northwest A&F University, Yangling 712100, China; 2 Yunnan Animal Science and Veterinary Institute, Jindian, Panlong District, Kunming 650224, China

**Keywords:** *Lentinus edodes*, rumen microbiota, metabolomics, immune enhancement, milk composition, oxidative stress

## Abstract

*Lentinus edodes* (LE) is a nutrient-rich medicinal fungus with potential applications in animal nutrition; however, its effects on dairy goats remain underexplored. This study investigated the impact of dietary LE addition on rumen microbiota, metabolic profiles, serum immunity, and milk quality in lactating dairy goats. Twenty Saanen goats were randomly assigned to a control group (CON, basal diet) or an LE group (basal diet + 25 g *Lentinus edodes*) for 56 days. Rumen fluid, serum, and milk samples were analyzed using 16S rDNA sequencing, metabolomics, and biochemical assays. The results showed that the addition of LE altered the microbial composition, decreasing the abundance of fibrobacterial flora and *Treponema* (*p* < 0.05) while increasing VadinHA49. Metabolomic analysis revealed elevated fumaric acid, lysophospholipids (LysoPE, LysoPG), and D-quinic acid in the rumen (*p* < 0.05). Serum immunoglobulin A (IgA), immunoglobulin G (IgG), total antioxidant capacity (T-AOC), superoxide dismutase (SOD), catalase (CAT), and tumor necrosis factor-α (TNF-α) levels were significantly enhanced (*p* < 0.05). The milk somatic cell count (SCC) was reduced by 52.8% (*p* = 0.005) at day 56, and protein, fat, and total solids increased significantly (*p* < 0.05). Enrichment of functional metabolites such as D-arabitol and D-tryptophan in milk further highlighted LE’s role in improving product value. These findings demonstrate that LE optimizes rumen flora, enhances antioxidant and immune functions, and improves milk quality, supporting its use as a functional feed additive for dairy goats.

## Simple Summary

*Lentinus edodes* (LE) is a nutrient-rich fungus, but its effects on dairy goats remain underexplored. In this study, we attempted to elucidate the specific effects of LE on the organic health and milk quality of dairy goats and to further investigate the mechanism of action of LE effects on dairy goats. Our results show that LE helps to optimize rumen flora, increase antioxidant capacity and immunity levels, and improve milk quality.

## 1. Introduction

Dairy goats have been an important part of animal husbandry for thousands of years, and the earliest known record of goat domestication is in the highlands of western Iran [1]. Goats are widely farmed around the world due to their excellent environmental adaptability. With the increasing demand for diversified dairy products, the scale farming of dairy goats has been expanding in recent years [2,3]. Human consumption of goat milk has a long history, and the world production of goat milk has been increasing in recent decades, and is expected to increase to 9.7 Mt by 2030 [4]. Goat milk contains smaller fat globules and casein micelles, and higher concentrations of short- and medium-long chain fatty acids compared to cow’s milk. As a result, the beneficial components of goat milk are more readily absorbed by the body and have a lower allergenicity. Goat milk has thus gained market acceptance as one of the important choices for animal dairy products [5,6].

*Lentinus edodes* (LE) is a medicinal and edible fungus that has attracted attention for its rich bioactive constituents, including polysaccharides, phenolic compounds, and ergosterol [7,8,9]. Numerous studies have highlighted its antioxidant, immunomodulatory, and antimicrobial properties in monogastric animal models. For example, in broiler chickens, addition with LE improved growth performance and cecum microbiota diversity [10], while in hypercholesterolemic rats, LE improved lipid metabolism and liver function [11]. Notably, in mouse models, Lentinus edodes polysaccharides activate macrophage-mediated immune responses and reduce oxidative stress [12]. However, less research is currently available for ruminants, with studies currently available only in horses [13] and calves [14]. Despite the differences in the model animals used in the studies, the positive effects of LE on the health of animals have been confirmed. Based on this, exploring the potential effects of LE on the production performance of dairy goats shows a positive research prospect.

Here, we hypothesized that dietary addition of LE could modulate the composition of the rumen flora and increase the production of beneficial metabolites, which in turn would improve systemic antioxidant capacity and milk quality in dairy goats. Therefore, the primary objective of this study was to evaluate the impact of LE addition on the rumen flora, serum antioxidant and immune profiles, and milk quality in dairy goats. Specifically, we aimed to determine whether LE could enhance the production of beneficial metabolites, improve antioxidant capacity, and positively influence milk composition and somatic cell count (SCC). Our findings provide novel insights into the potential of LE as a multi-target feed additive that integrates microbial ecology, host physiology, and dairy quality in ruminants.

## 2. Materials and Methods

### 2.1. Ethics Statement

The procedures of this study were approved by the Animal Ethical and Welfare Committee of Northwest A&F University (Yangling, China, Approval No. DK2022008, Approval Date: 2022) and were in accordance with the university’s guidelines for animal research.

Animals were housed at Antelope Qing Herding Co., Ltd. (Honghe Prefecture, China).

### 2.2. Animals, Experimental Design and Feeding

After 2 weeks of adaption, 20 healthy Saanen dairy goats with uniform body size, close lactation performance, and about 60 ± 5 days of milk production in the first kidding were selected and randomly divided into two groups of 10 goats each: a CON group (fed a basal diet) and an LE group (fed a basal diet + 25 g of LE). The average particle size of LE is about 0.5 mm, and the feeding method is to mix it well with the basal diet and then give it to the dairy goats. The chemical composition and nutritional levels of a basal diet are shown in Table 1. Nutritional levels of LE are shown in Table S1.

All dairy goats were fed twice daily at 07:00 and 18:00, respectively. Milking was performed daily at 06:30 and 16:30 using a portable milking machine. The official test lasted 56 days.

In this study, the experimental animals were divided into LE and CON groups, and six dairy goats were randomly selected from each group (12 in total). Their rumen fluid samples were subjected to 16S rDNA gene sequencing, and metabolomics analyses of the rumen fluid and the corresponding goat’s milk samples were carried out simultaneously. It is worth noting that all the rest of the assays in the experimental design (including but not limited to serum biochemical indexes, rumen fermentation parameters, etc.) were measured in an intact group of 20 experimental animals.

### 2.3. Feed Sample Collection and Analysis

Before and after each feeding, feed intake and residual amount were weighed to calculate feed intake of the goats. On day 56, feed samples and LE samples were collected and analyzed for the following: dry matter (DM, method 930.15), crude protein (CP, method 976.05), calcium and phosphorus (method 935.13), fat (EE, method 920.39), and ash (method 935.13) [15]. In addition, neutral detergent fiber (NDF, prepared using amylase and sulfite treatment) and acid detergent fiber (ADF) contents of the samples were determined using the method of Van Soest et al. [16].

### 2.4. Rumen Fluid Analysis

On day 56 of the experiment, fresh rumen fluid samples were taken from the rumen of each goat through a rumen catheter. This was carried out by opening the goat’s mouth and inserting the head of a sampler connected to a vacuum pump, a hard plastic tube 3 cm in diameter and 90 cm long, into the esophagus, with the vacuum pressure set at 65 kPa. To minimize salivary contamination, approximately 30 mL of rumen fluid was discarded prior to sampling. The sample was immediately filtered through sterilized four-ply gauze, resulting in a final sample of approximately 50 mL of rumen fluid. The collected samples were assayed for ammoniacal nitrogen (NH_3_-N) concentration by colorimetric method and volatile fatty acids (VFA) using gas chromatography (Agilent, 7890, Santa Clara, CA, USA).

### 2.5. 16S rDNA Amplicon Sequencing of Rumen Fluid

To analyze 16S rDNA, rumen fluid from six randomly selected dairy goats was used to extract genomic DNA using the OMEGA Soil DNA Kit (D5625-01, Omega Bio-Tek, Norcross, GA, USA) and tested for purity and concentration. PCR amplification of the selected V3-V4 variable region was conducted using specific primers (338F, 5′-ACTCCTACGGGAGGCAGCAG-3′; 806R,5′-GGACTACHVGGGTWTCTAAT-3′). The PCR products were detected by 2% agarose gel electrophoresis, and after recovery of the target fragments, gel recovery was carried out using the Quant-iT PicoGreen dsDNA Assay Kit (Thermo Fisher Scientific, Waltham, MA, USA). Based on the preliminary quantification results of electrophoresis, the PCR products were quantified using a Microplate reader (BioTek, FLx800) fluorescence quantification system and mixed as required by the sequencing volume of the samples. Library construction was performed using the Illumina TruSeq Nano DNA LT Library Prep Kit (Illumina, San Diego, CA, USA), and the constructed libraries were quality controlled by the Agilent Bioanalyzer 2100 (Agilent, Santa Clara, CA, USA) and Promega QuantiFluor (Promega, Madison, WI, USA). After the libraries were qualified, they were put on the machine for sequencing (Zhongke New Life, Shanghai, China).

Raw sequencing data were in FASTQ format. Paired-end reads were then preprocessed using cutadapt software (Cutadapt v4.9) to detect and cut off the adapter. After trimming, paired-end reads were filtered for low-quality sequences, denoised, merged, and we detected and cut off the chimera reads using DADA2 (DADA2 1.26.0) with the default parameters of QIIME2. Finally, the software output the representative reads and the ASV abundance table. The representative read of each ASV was selected using the QIIME 2 package. All representative reads were annotated and blasted against the Silva database Version 138 using classify-sklearn with the default parameters. We calculated the α, β diversity index with QIIME2 software (QIIME2 2024.5). Alpha diversity was used to analyze the diversity of microbial communities within the sample. The richness and diversity of microbial communities in a sample can be reflected through the diversity analysis of a single sample (alpha diversity), including the use of the species accumulation box chart, species diversity curve, and a series of statistical analysis indexes to evaluate the differences of species richness and diversity of microbial communities in each sample. Beta diversity is a comparative analysis of microbial community composition of different samples. LEfSe (linear discriminant analysis effect size, linear discriminant analysis, and influence factor) can be used to find the species characteristics that can best explain the differences between groups in two or more groups of samples, and the influence degree of these characteristics on the differences between groups. Using the inter-group difference test method, according to the community abundance data obtained, using the strict statistical method, the species of two groups/multiple groups of sample microbial communities were hypothesis tested. The significance level of species abundance difference was evaluated, and the species information with significant difference between the two groups was obtained. The STAMP difference analysis is used to compare the species abundance between two groups of samples (Wilcox test analysis). Through this analysis, the species with significant difference can be obtained.

### 2.6. Serum Indices Analysis

On day 56 of the experiment, 10 mL of venous blood was collected from each goat, followed by centrifugation at 35,000 r/min for 5 min, and the serum was separated and stored at −80 °C. The levels of immunoglobulin A (IgA), immunoglobulin G (IgG), immunoglobulin M (IgM), total antioxidant capacity (T-AOC), superoxide dismutase (SOD), catalase (CAT), glutathione peroxidase (GSH-Px), and tumor necrosis factor-α (TNF-α) in serum were determined using the enzyme-linked immunosorbent assay (ELISA) according to the instructions provided by the kit manufacturer (Jiangsu Enzyme-linked Immunoassay Company, Yancheng, China). In addition, an automatic biochemical analyzer (BK-400, Northwest A&F University, Yangling, China) was used to detect serum biochemical indices, including aminotransferase (ALT), aspartate aminotransferase (AST), alkaline phosphatase (ALP), albumin (ALB), cholesterol (CHO), D-3-hydroxybutyric acid (D-3-HBA), antithymocyte globulin (ATG), total protein (TP), α-hydroxybutyric dehydrogenase (α-HBDH), non-esterified fatty acid (NEFA), and beta-hydroxybutyrate (β-Hb).

### 2.7. Goat Milk Analysis

On days 0, 14, 28, 42, and 56 of the experiment, 50 mL of goat’s milk per dairy goat was collected in the morning and at the next collection, respectively, and mixed in the ratio of 6:4 to form a goat’s milk sample. These mixed samples were sent to Xi’an General Animal Husbandry Station in Shaanxi, China for determination of milk fat, milk protein, lactose, total milk solids, and somatic cell count (SCC).

### 2.8. Metabolomic Analysis of Goat Milk and Rumen Fluid

The six randomly selected goat milk samples and rumen fluid samples were thawed gradually on ice. A 100 μL aliquot of each sample was transferred to a 1.5 mL centrifuge tube, followed by the addition of 300 μL methanol. The samples were vortexed to ensure thorough mixing. Subsequently, they were sonicated at 4 °C for 30 min using a PS-80A sonicator (Shanghai Ledon Industry). After sonication, the samples were incubated at −40 °C for 1 h, vortexed for 30 s, and then allowed to stand at 4 °C for an additional 30 min. The supernatant was carefully collected into a new centrifuge tube, followed by an additional incubation at −40 °C for 1 h. The samples were then centrifuged at 4 °C for 15 min at 12,000 rpm. The resulting supernatant (200 μL) was mixed with 5 μL of the internal standard (0.20 mg/mL dichlorophenylalanine), thoroughly vortexed, and transferred into an injection vial for further analysis.

Metabolite profiling was performed using an LC-MS system (Waters, UPLC; Thermo Q Exactive, Thermo Fisher Scientific, Waltham, MA, USA). Chromatographic separation was carried out on an ACQUITY UPLC HSS T3 column (2.1 × 100 mm, 1.8 μm). The column temperature was set to 40 °C with a flow rate of 0.300 mL/min. The mobile phase consisted of (A) water with 0.05% ammonium and (B) acetonitrile. The injection volume was 5 μL, and the automatic injector temperature was maintained at 4 °C.

For positive ion mode (ESI+), the heater temperature was set to 300 °C, sheath gas flow rate to 45 arb, auxiliary gas flow rate to 15 arb, sweep gas flow rate to 1 arb, and spray voltage to 3.0 kV. The capillary temperature was 350 °C, and the S-lens RF level was 30%. For negative ion mode (ESI-), the heater temperature remained at 300 °C, sheath gas flow rate at 45 arb, auxiliary gas flow rate at 15 arb, and sweep gas flow rate at 1 arb. The spray voltage was adjusted to 3.2 kV, the capillary temperature was 350 °C, and the S-lens RF level was set to 60%.

Raw data files were imported into Compound Discoverer 3.1 software (Thermo Fisher Scientific) for spectral processing and database search to perform both qualitative and quantitative metabolite analysis. Multivariate statistical analysis, including principal component analysis (PCA) and partial least squares discriminant analysis (PLS-DA), was performed using SIMCA software (Version 14.1, Sartorius Stedim Data Analytics AB, Umeå, Sweden) to identify metabolic differences between groups. Hierarchical clustering analysis (HCA) and metabolite correlation analyses were employed to explore the relationships among samples and between metabolites. Metabolic pathways were annotated using the Kyoto Encyclopedia of Genes and Genomes (KEGG) Pathway Database (http://www.kegg.jp/kegg/pathway.html, accessed on 2 April 2024) to elucidate the biological significance of metabolite interactions.

### 2.9. Data Analysis and Statistics

Continuous variables including rumen fermentation parameters, serum biochemical indices, milk composition, and SCC were assessed for normality using Shapiro-Wilk tests. Normally distributed data were analyzed by independent two-tailed *t*-tests with homogeneity of variance confirmed by Levene’s test, while non-parametric Wilcoxon rank-sum tests were applied to skewed distributions (SPSS 19.0; IBM Corp., Armonk, NY, USA). SCC, feed intake, and serum antioxidant and immune indices were visualized using GraphPad Prism 10.1.2. Interdomain correlations integrating rumen microbiota (genus-level), rumen metabolites, serum immunology and antioxidant markers, and milk metabolic profiles were examined through Spearman’s rank analysis using OmicStudio (v1.8.3). Significant associations were defined as |ρ| > 0.5 with FDR-adjusted *p* < 0.05.

## 3. Results

### 3.1. Effect of LE on Feed Intake and Rumen Fermentation Parameters in Dairy Goats

**Figure 1:**
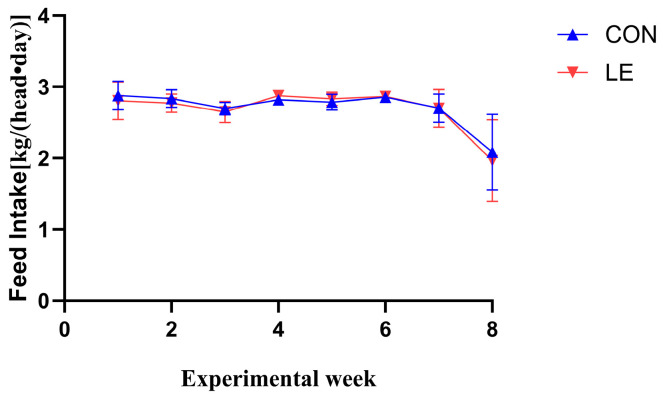
Daily feed intake of dairy goats in CON and LE groups during the 56-day trial (*n* = 10/group). CON = basal diet; LE = basal diet + 25 g *Lentinus edodes*. Data are expressed as mean ± SEM. No significant difference is indicated.

No significant difference in feed intake was observed between the CON and LE groups (*p* > 0.05, Figure 1).

The NH_3_-N content in the rumen fluid of the LE group showed a decreasing trend compared to that of the CON group (8.72 vs. 6.52 mg/dL, *p* = 0.056; Table 2), though it did not reach the significance threshold (*p* < 0.05). However, LE treatment had no significant effect on rumen concentrations of acetate, propionic acid, isobutyric acid, butyric acid, isovaleric acid, volatile fatty acids, and acetate/propionic acid ratio (*p* > 0.05).

### 3.2. Effect of LE on Rumen Flora in Dairy Goats

To investigate the effect of LE treatment on rumen microbes of dairy goats, we performed 16S rDNA sequencing of rumen fluid samples on day 56. The rarefaction curve was flat, indicating a reasonable amount of sequencing data. The two groups did not show significant differences in Shannon, Simpson, and Chao1 indices. In addition, β-diversity demonstrated no significant difference in the community composition of rumen flora of dairy goats in the two groups (*p* > 0.05, Figures S1 and S2).

**Figure 2:**
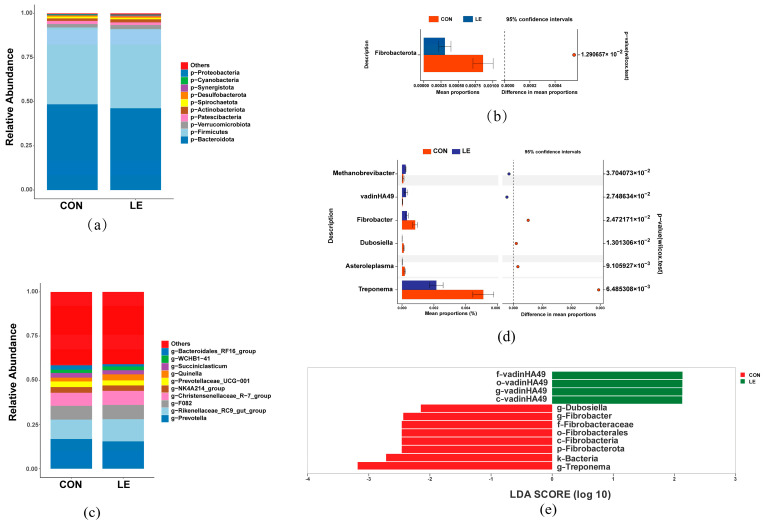
Rumen microbiota composition and differential taxa analysis (*n* = 6/group). (**a**) Relative abundance of the top 10 bacterial phyla. (**b**) Differences between bacterial taxa CON and LE groups at the phylum level. (**c**) Relative abundance of the top 10 bacterial genera. (**d**) Differences between bacterial taxa CON and LE groups at the genus level. (**e**) Linear discriminant analysis (LDA) and effect size (LEfSe) analyses were performed to identify bacterial taxa represented at different taxonomic levels of difference in the CON and LE groups. CON = basal diet; LE = basal diet + 25 g *Lentinus edodes*.

We analyzed the relative abundance of microorganisms labeled as ASVs at the phylum and genus levels. At the phylum level, Bacteroidota had the highest abundance, representing 48.52% and 46.28% of dairy goats in the CON and LE groups, respectively. Other phylum such as Firmicutes (43.52%, 44.82%), Verrucomicrobiota (1.96%, 2.29%), Patescibacteria (1.74%, 1.51%), Actinobacteriota (1.43%, 1.77%), and Spirochaetota (1.03%, 0.89%) were closer in abundance in both groups, and the abundance was greater than 1.00% in at least one group (Figure 2a).

Stamp analysis showed that the abundance of Fibrobacterota was significantly reduced by LE treatment (*p* = 0.0161, Figure 2b).

At the genus level, the top 9 dominant bacteria in the CON and LE groups were *Prevotella* (16.78%, 15.44%), *Rikenellaceae_RC9_gut_group* (11.00%, 12.55%), *F082* (7.79%, 8.09%), *Christensenellaceae_ R-7_group* (7.35%, 7.93%), *NK4A214_group* (3.21%, 3.06%), *Prevotellaceae_UCG-001* (3.20%, 2.82%), *Quinella* (2.15%, 3.44%), *Succiniclasticum* (2.66%, 2.36%), *WCHB1-41* (1.84%, 2.15%), and *Bacteroidales_RF16_group* (2.50%, 1.23%, Figure 2c).

LE significantly increased the relative abundance of *Methanobrevibracter* (*p* = 0.045) and *VadinHA49* (*p* = 0.0338), while significantly decreasing *Asteroleplasma* (*p* = 0.0401), *Fibrobacter* (*p* = 0.0303), *Dubosiella* (*p* = 0.0165), and *Treponema* (*p* = 0.00433, Figure 2d).

Linear discriminant analysis (LDA) effect size (LEfSe) was used to estimate the impact of the abundance of each species on the differential effect (logarithmic LDA score > 2.0). The results demonstrated that Fibrobacterota was significantly enriched at the phylum level, and *Treponema*, *Fibrobacter*, and *Dubosiella* were significantly enriched at the genus level of rumen bacteria from dairy goats in the CON group. VadinHA49 was significantly enriched at the level of phylum, order, family, and genus of rumen bacteria in dairy goats of the LE group (Figure 2e).

### 3.3. Effect of LE on Rumen Metabolites in Dairy Goats

Using a VIP threshold of >1 and *p* < 0.05, we identified 32 differential metabolites between the LE group and the CON group, 17 positively ionized metabolites and 15 negatively ionized metabolites (Table S2).

**Figure 3:**
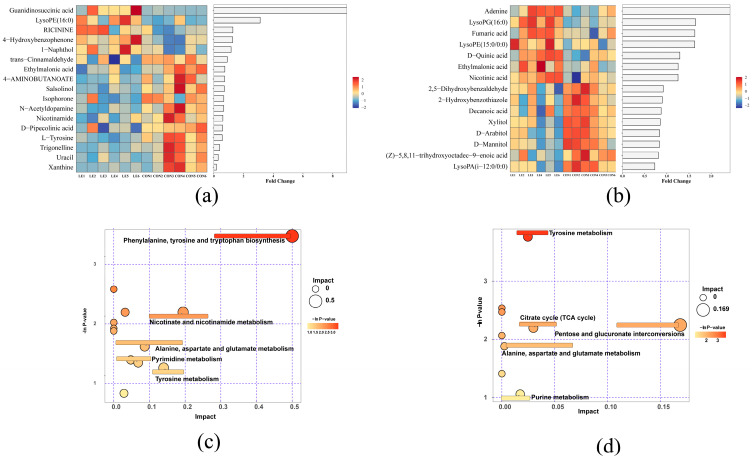
Rumen metabolome profiles and KEGG pathway enrichment (*n* = 6/group). (**a**,**b**) Heatmaps and differential fold histograms of differential metabolites in positive (POS) and negative (NEG) ion modes (VIP > 1, *p* < 0.05). FC refers to the differential expression multiple of this metabolite between the two groups. Bar lengths represent log2 fold change (log2FC) values, with red indicating upregulation and blue indicating downregulation. (**c**,**d**) KEGG pathway enrichment map of metabolites in the rumen. The *x* axis represents the pathway impact, and the *y* axis represents the pathway enrichment. A larger size and darker color represent higher pathway enrichment and higher pathway impact value, respectively. CON = basal diet; LE = basal diet + 25 g *Lentinus edodes*.

LE treatment significantly increased the relative concentrations of guanidinosuccinic acid, LysoPE (16:0), LysoPG (16:0), fumaric acid, LysoPE (15:0/0:0), and D-quinic acid in ruminal fluid (*p* < 0.05), while significantly decreasing the relative concentrations of xanthine, uracil, trigonelline, and L-tyrosine (*p* < 0.05, Figure 3a,b).

The *p*-values and impact values of the pathway analyses demonstrated significant differences (*p* < 0.05) in metabolites between the CON and LE groups. The results demonstrated that major metabolic pathways such as phenylalanine, tyrosine and tryptophan biosynthesis, nicotinic acid and nicotinamide metabolism, tyrosine metabolism, pentose and glucuronide interconversion, alanine, aspartate and glutamate metabolism, pyrimidine metabolism, arginine and proline metabolism, butyric acid metabolism, and the citric acid cycle (TCA cycle) were enhanced by the addition of 25 g of LE (Figure 3c,d).

### 3.4. Effect of LE on Serum Indices of Dairy Goats

LE treatment had no significant effect on serum biochemical indices, including ALT, AST, ALP, ALB, CHO, D-3-HBA, TG, TP, α-HBDH, NEFA, and β-HB in dairy goats (*p* > 0.05, Table 3).

**Figure 4:**
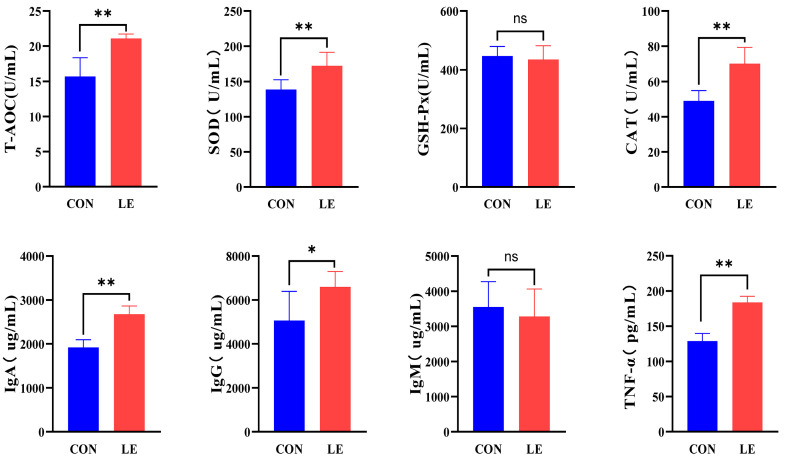
Serum antioxidant and immune indices (*n* = 10/group). T-AOC = total antioxidant capacity; SOD = superoxide dismutase; GSH-Px = glutathione peroxidase; CAT = catalase; IgA = immunoglobulin A; IgG = immunoglobulin G; IgM = immunoglobulin M; TNF-α = tumor necrosis factor-α. Data are expressed as mean ± SEM. Significance: ns (*p* > 0.05), * (*p* < 0.05), ** (*p* < 0.01). CON = basal diet; LE = basal diet + 25 g *Lentinus edodes*.

After LE treatment, the serum levels of IgA, T-AOC, SOD, CAT, and TNF-α in dairy goats were significantly higher than those in the CON group (*p* < 0.01), and the IgG content was significantly higher than that in the CON group (*p* < 0.05), whereas the IgM and GSH-Px contents did not show any significant difference (*p* > 0.05, Figure 4).

### 3.5. Effect of LE on Lactation Performance and Milk Quality of Dairy Goats

There was no significant difference (*p* > 0.05) in the mean daily milk yield after LE treatment compared to the CON group. Milk fat, milk protein, and total solids content in goat’s milk were significantly higher (*p* < 0.05) after LE treatment, while lactose content did not undergo a significant change (*p* < 0.05, Table 4).

**Figure 5:**
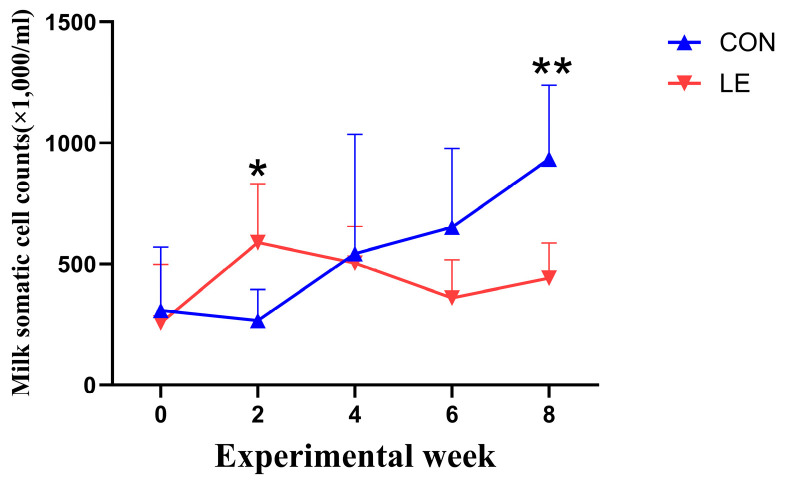
Analysis of somatic cell count (SCC) in goat’s milk (*n* = 10/group). CON = basal diet; LE = basal diet + 25 g *Lentinus edodes*. Data are expressed as mean ± SEM. Significance: * 0.01 < *p* ≤ 0.05; ** *p* < 0.01.

Compared with the CON group, although the mean SCC of the LE group did not change significantly (*p* > 0.05, Table 4), the SCC of the LE group changed at a specific time after LE treatment. Specifically speaking, although the SCC in the LE group was significantly higher than that in the CON group on day 14 (*p* = 0.016), the trend of elevated SCC was reversed thereafter. On day 56, the SCC in the LE group was significantly lower than that in the CON group (*p* = 0.005, Figure 5).

To analyze metabolite differences in goat milk between the LE and CON groups, we applied a VIP threshold value greater than 1 (*p* < 0.05). This analysis identified 50 metabolites with significant differences, including 20 positively ionizing metabolites and 30 negatively ionizing metabolites (Table S3).

**Figure 6:**
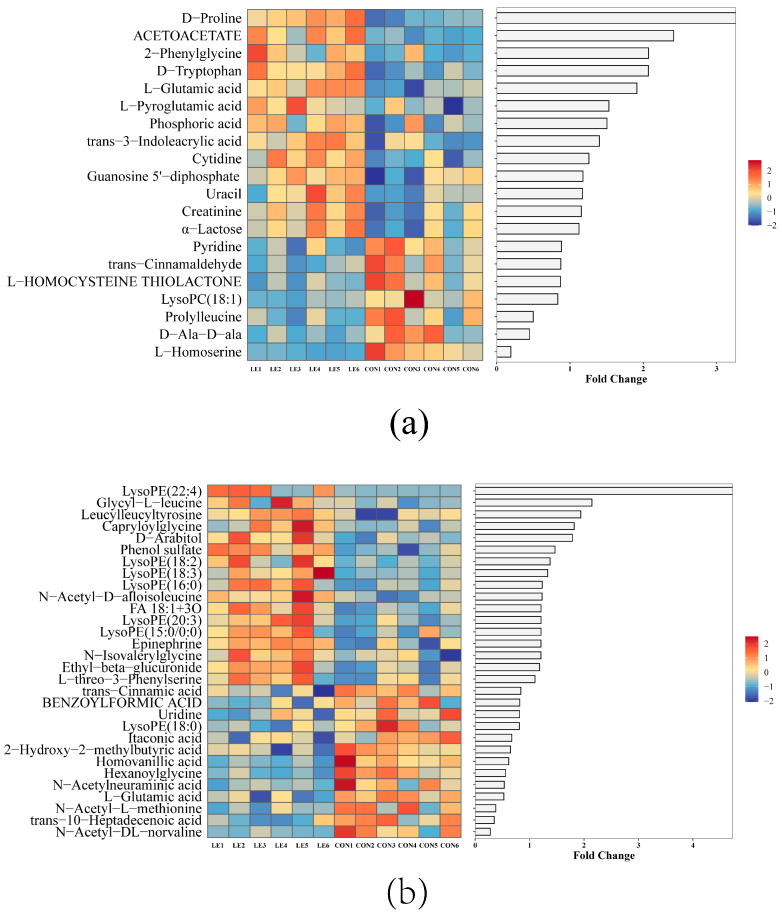
Analysis of differential metabolite expression in goat’s milk in LE and CON groups (*n* = 6/group). FC refers to the differential expression multiple of this metabolite between the two groups. Bar lengths represent log2 fold change (log2FC) values, with red indicating upregulation and blue indicating downregulation. CON = basal diet; LE = basal diet + 25 g *Lentinus edodes*. (**a**) Differential fold and heat maps of metabolite expression in goat’s milk between LE and CON groups in positive ion mode; (**b**) Differential fold and heat maps of metabolite expression in goat’s milk between LE and CON groups in negative ion mode.

LE significantly enhanced LysoPE (22:4), D-proline, acetoacetate, glycyl-L-leucine, 2-phenylglycine, D-tryptophan, leucyl-leucyl-tyrosine, L-glutamic acid, capryloyl glycine and D-arabitol in goat milk. Yet we found significantly lower L-homoserine, N-acetyl-DL-norvaline, trans-10-heptadecenoic acid, N-acetyl-L-methionine, D-ala-D-ala, prolylleucine, and L-glutamic acid relative concentrations (*p* < 0.05, Figure 6a,b).

### 3.6. Correlation Analysis

**Figure 7:**
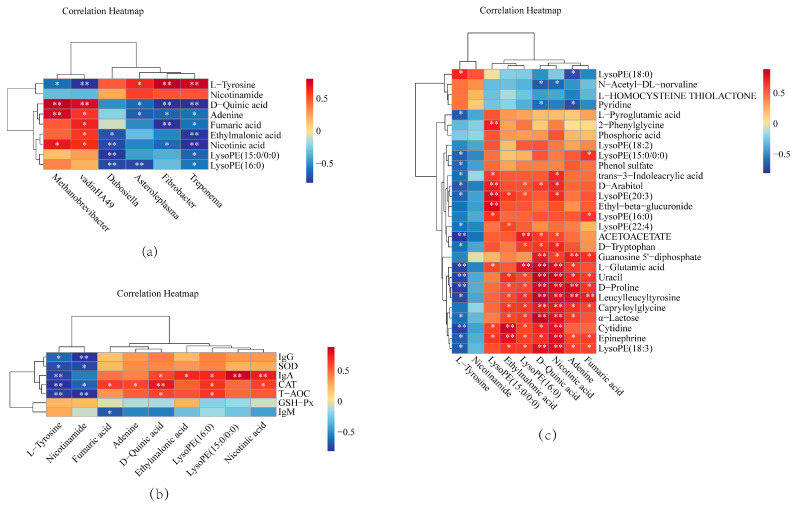
Spearman correlation analysis. (**a**) Correlation analysis between rumen flora and rumen metabolites. (**b**) Correlation analysis of rumen metabolites with serum immune and antioxidant levels. (**c**) Correlation analysis between rumen metabolites and goat milk metabolites. Each lattice represents the Spearman correlation coefficient between rows and columns. Red color indicates positive correlation while blue color indicates negative correlation. Significance: * 0.01 < *p* ≤ 0.05; ** *p* < 0.01. CON = basal diet; LE = basal diet + 25 g *Lentinus edodes*.

In order to investigate the possible reasons for the changes in the bodies of dairy goats after feeding LE, we investigated the relationships between rumen flora and rumen metabolites, rumen metabolites and serum immune and antioxidant levels, and rumen metabolites and goat milk metabolites using Spearman’s rank correlation analysis. The results demonstrated that vadinHA49 was negatively correlated with L-tyrosine and positively correlated with D-quinic acid, adenine, fumaric acid, ethylmalonic acid, and nicotinic acid (Figure 7a). L-tyrosine and nicotinamide in the rumen were negatively correlated with IgG, SOD, IgA, CAT, and T-AOC in serum, yet D-quinic acid was positively correlated with IgA, CAT, and T-AOC in serum (Figure 7b). L-tyrosine in the rumen was positively correlated with LysoPE (18:0) in goat’s milk and negatively correlated with 17 metabolites, including D-proline, L-glutamic acid, D-tryptophan, and D-arabitol (Figure 7c).

## 4. Discussion

The present study showed that the addition of LE had a tendency to reduce rumen NH_3_-N concentrations (*p* = 0.056), but did not reach the significance threshold. This result is in line with the findings that the combination of probiotics and phytopolysaccharides can reduce rumen NH_3_-N concentration in lambs [17,18,19]. The possible mechanism is that the phenolic components of LE inhibit the activity of highly ammonia-producing bacteria [20,21], thereby reducing NH_3_-N production. Notably, although volatile fatty acids (VFA) and major components such as acetic acid and propionic acid did not change significantly (*p* > 0.05), rumen flora structure was specifically altered. For example, LE significantly reduced the relative abundance of Fibrobacterota and *Treponema* (*p* < 0.05). Fibrobacterota (phylum Fibrobacteria) was significantly reduced in abundance (*p* = 0.0161), and its reduction as a fiber-degrading bacterium may partly account for the decrease in NH_3_-N [22], and *Treponema* may be associated with cardiovascular disease risk [23]. Meanwhile, there was a significant increase in the abundance of VadinHA49 (belonging to the phylum Planctomycetota) which, along with Bacteroidota and several other phyla, has been identified as one of the most versatile phyla for the degradation of a wide range of biopolymers of cellulosic and non-cellulosic origin [24], and which encodes a carbohydrate-active enzyme that may promote fiber degradation and propionic acid production [25,26], but propionic acid concentration did not change significantly in the experiments (*p* > 0.05), suggesting that its metabolic fluxes may be diverted by other pathways, such as the TCA cycle. Although there was no significant change in α-diversity and β-diversity (*p* > 0.05), which is different from the findings of GUO et al. [10], we believe that this difference may be caused by the different digestion patterns of broilers and dairy goats. However, it should not be overlooked that the adjustment of specific flora may indirectly affect host health through metabolic pathways.

Metabolomics analysis showed that LE significantly upregulated fumaric acid and lysophospholipid (LysoPE, LysoPG) concentrations (*p* < 0.05), while decreasing L-tyrosine and nicotinamide levels (*p* < 0.05). These changes are closely related to the functional remodeling of the colony: proliferation of VadinHA49 may promote fumaric acid accumulation by enhancing TCA cycle activity, which acts as an antioxidant to inhibit DNA damage repair pathways (e.g., TLS) [27], thus enhancing the organism’s resistance to stress. The reduction in Fibrobacterota may have reduced the activity of the phenylalanine-tyrosine metabolic pathway and reduced the production of potentially harmful metabolites (e.g., phenol derivatives) [28], consistent with the downregulation of L-tyrosine. In contrast, LysoPE improves the body’s state by modulating the inflammatory response and lipid metabolism [29,30,31,32]. The reduction of *Treponema* (*p* = 0.00433) may reduce the risk of lipopolysaccharide (LPS) release [33], which synergizes with the anti-inflammatory effects of LysoPE to alleviate host inflammation. Furthermore, elevated D-quinic acid may mitigate the negative effects of a high-fat diet by promoting NAD+ synthesis [34].

LE significantly elevated serum IgA, IgG, T-AOC, SOD, and TNF-α levels (*p* < 0.05), whereas liver function parameters (ALT, AST) and lipid metabolism parameters (CHO, TG) were not affected (*p* > 0.05). This result suggests that LE polysaccharides may promote immunoglobulin secretion through activation of macrophages [35], whereas the anti-inflammatory effect of LysoPG (16:0) may indirectly support immune homeostasis, fumaric acid, and D-quinic acid, and that by scavenging free radicals and activating the AMPK pathway [27,34], together with elevated SOD and CAT, oxidative damage was mitigated. The absence of differences in serum IgM and GSH-Px (*p* > 0.05) may reflect selective modulation of specific immune pathways by LE rather than full activation. These results are in agreement with the positive results obtained by Nisar et al. [11] and Muszyńska et al. [14], thus providing further evidence of the positive effects of LE on the animal body.

LE significantly increased protein, fat and total solids in goat’s milk (*p* < 0.05), while there was no significant effect on lactose (*p* > 0.05). This result correlates with the high protein properties of LE and the potential facilitation of rumen microbial protein synthesis [8,36], and although milk yield did not change significantly (*p* > 0.05), the somatic cell count (SCC) in milk was significantly lower (*p* = 0.005) at day 56, suggesting that LE may improve mammary health.

Milk metabolomics further revealed that elevated D-arabitol and D-tryptophan (*p* < 0.05) may enhance the added value of dairy products by regulating blood glucose [37,38,39] and inhibiting pathogenic bacterial biofilms [40]. The decrease in rumen L-tyrosine was negatively correlated (*p* < 0.05) with the increase in D-proline in milk, suggesting that cross-tissue redistribution of amino acid metabolism may be realized through the colony-host axis [41,42,43,44,45].

Spearman’s correlation analysis indicated that the association of rumen flora (e.g., *VadinHA49*) with metabolites (e.g., D-quinic acid, fumaric acid) may indirectly affect milk composition through modulation of host immune and antioxidant pathways. For example, a decrease in rumen L-tyrosine was negatively correlated (*p* < 0.05) with an increase in milk D-proline, suggesting that rumen metabolism may influence milk quality through amino acid conversion. In addition, the significant correlation (*p* < 0.01) between serum IgA, T-AOC, and rumen metabolites further supports the overall regulation of the rumen-host-milk axis. Although the activation of some metabolic pathways (e.g., pyrimidine metabolism) did not directly correlate with significant phenotypic changes, its potential function needs to be verified in long-term studies.

Although LE did not have a significant effect (*p* > 0.05) on some indicators (e.g., total VFA, lactose content, serum TG), these results are still informative. For example, the stability of rumen VFA composition may indicate that LE acted mainly through colony structural adjustment rather than fermentation pattern alteration; and the absence of differences in lactose content suggests that LE had limited effects on mammary glucose metabolism. In addition, the stability of serum biochemical indices (e.g., ALT, AST) further demonstrated the safety of LE and provided a basis for its long-term application as a feed additive.

There is a lack of studies on the direct addition of LE to dairy goat diets, and the results are not directly comparable with those reported previously. We believe that the effect of LE on the organism of dairy goats stems not only from the dosage effect, but also due to the persistence of the addition. Limitations of our study are mainly that this study was a small project involving 20 goats, which may limit the statistical validity of the data. Future studies should further investigate the optimal level of LE addition and its different effects on different stages of the lactation cycle to optimize production benefits. It should also be gradually extended to other economic livestock species such as beef cattle and sheep to promote the development of additives for functional feeds.

## 5. Conclusions

In this study, we systematically evaluated the effects of dietary LE on rumen flora, metabolic profiles, serum immunity, and milk quality of dairy goats, and came to the following conclusions: in terms of rumen regulation, LE significantly reduced (*p* < 0.05) the abundance of Fibrobacterota and *Treponema* in the rumen, while promoting the proliferation of fiber-degrading bacteria *VadinHA49* proliferation, and upregulated the concentrations of metabolites such as fumaric acid and LysoPE/LysoPG (*p* < 0.05), suggesting that LE can enhance fiber utilization efficiency by remodeling the structure of the rumen flora and metabolic patterns. At the level of body health, LE significantly enhanced serum immunoglobulin (IgA, IgG) levels and activities of total antioxidant capacity (T-AOC), superoxide dismutase (SOD), and catalase (CAT) (*p* < 0.05), and did not negatively affect liver function and lipid metabolism indexes, such as aminotransferase (ALT), aspartate aminotransferase (AST), and cholesterol (CHO) (*p* > 0.05), confirming its dual efficacy of enhancing immunity and alleviating oxidative stress. In terms of milk quality optimization, LE significantly reduced the milk somatic cell count (SCC) (*p* = 0.005), significantly increased milk protein rate, fat rate, and total solids content (*p* < 0.05), and enriched functional metabolites such as D-arabitol and D-tryptophan (*p* < 0.05), which provided a theoretical basis for the development of high-value-added dairy products.

## Figures and Tables

**Table 1:** Ingredient and nutritional composition of the basal diet (air-dry basis).

Items	Content
Ingredient, %	
Yellow corn	30.00
Soybean meal	5.00
Rapeseed meal	4.50
Bran	7.00
Corn silage	20.00
Oat grass	10.00
Dried alfalfa	7.50
Peanut seedlings	12.50
Salt	0.75
Dicalcium phosphate	0.75
Vitamin-mineral premix ^1^	2.00
Total	100.00
Nutrient levels	
Crude protein	12.64
Neutral detergent fiber	33.66
Acid detergent fiber	22.63
Ethyl extract	1.64
Crude ash	12.00
Calcium	1.05
Phosphorous	0.49

^1^ The premix provided the following per kg of diets: VA, 350,000 IU; VD3, 93,750 IU; VE, 938 mg; VK3, 63 mg; VB1, 62 mg; VB2, 188 mg; niacin, 750 mg; pantothenic acid, 500 mg; VB6, 62 mg; biotin, 3.7 mg; folic acid, 38 mg; VB12, 0.7 mg; Se, 18 mg; Zn, 3000 mg; I, 23 mg; Co, 30 mg; Mn, 2500 mg; Fe, 3240 mg; Cu 500 mg.

**Table 2:** Effect of LE on rumen fermentation parameters in dairy goats (*n* = 10/group).

Items	CON	LE	SEM	*p*-Value
Ammoniacal nitrogen (mg/dL)	8.72	6.52	1.104	0.056
Total VFA (mmol/L)	89.14	77.70	10.318	0.293
Individual VFA (mmol/L)				
Acetate	66.20	58.30	7.971	0.345
Propionate	13.20	10.86	1.542	0.159
Isobutyrate	0.88	0.77	0.110	0.373
Butyrate	7.20	6.25	0.919	0.325
Isovalerate	1.17	1.09	0.147	0.594
Valerate	0.49	0.43	0.050	0.282
Acetate/Propionate	5.06	5.38	0.286	0.283

VFA = volatile fatty acids. CON = basal diet; LE = basal diet + 25 g *Lentinus edodes*. SEM = standard error of the mean.

**Table 3:** Effect of LE on serum biochemical indices in dairy goats (*n* = 10/group).

Item	CON	LE	SEM	*p*-Value
ALT, U/L	17.17	18.22	1.936	0.600
AST, U/L	122.77	124.58	9.202	0.847
ALP, U/L	119.67	86.50	20.937	0.144
ALB, g/L	39.35	38.13	2.817	0.675
CHO, mmol/L	3.64	3.07	0.345	0.132
D3H, mmol/L	0.23	0.28	0.035	0.173
TG, mmol/L	0.31	0.37	0.054	0.296
TP, g/L	99.68	95.48	5.490	0.462
α-HBDH, mmol/L	299.83	329.00	27.026	0.316
NEFA, mmol/L	0.68	0.83	0.151	0.343
β-HB, mmol/L	0.28	0.26	0.060	0.752

ALT = aminotransferase; AST = aspartate aminotransferase; ALP = alkaline phosphatase; ALB = albumin; CHO = cholesterol; D-3-HBA = D-3-hydroxybutyric acid; ATG = antithymocyte globulin; TP = total protein; α-HBDH = α-hydroxybutyric dehydrogenase; NEFA = non-esterified fatty acid; β-Hb = beta-hydroxybutyrate. CON = basal diet; LE = basal diet + 25 g *Lentinus edodes*. SEM = standard error of the mean.

**Table 4:** Effects of LE on milk yield, somatic cell count (SCC), and milk composition in dairy goats (*n* = 10/group).

Item	CON	LE	SEM	*p*-Value
Milk yield (kg/(head·day)]	1.17	1.13	0.264	0.115
Somatic cell count (×1000/mL)	540.07	429.53	40.923	0.179
Composition (%)				
Fat	2.72	2.92	0.390	0.008
Protein	2.79	2.89	0.190	0.010
Lactose	4.60	4.54	0.022	0.232
Total solids content	10.39	10.65	0.451	0.004

CON = basal diet; LE = basal diet + 25 g *Lentinus edodes*. SEM = standard error of the mean.

## Data Availability

Follow-up research on this project is ongoing; please contact the corresponding author with reasonable requests.

## Supplementary Materials

The following supporting information can be downloaded at: https://www.mdpi.com/article/10.3390/ani15050676/s1. Figure S1: 16S rDNA Rarefaction Curve and Shannon Curve of rumen fluid of dairy goats; Figure S2: 16S rDNA microbial composition and diversity of the rumen fluid of dairy goats; Figure S3: Dairy goat rumen fluid metabolite quality test and volcano diagram; Figure S4: Dairy goat milk metabolite quality test and volcano diagram; Table S1: Nutritional level of *Lentinus edodes*; Table S2: Statistical results of differential metabolites in rumen fluid; Table S3: Statistical results of differential metabolites in goat milk.
