# Supplementary figures and images for: Effect of Dietary Addition of Lentinus edodes on Rumen Flora, Lactation, and Health of Dairy Goats
Source: Animals (Basel). 2025 Feb 26;15(5):676. doi: 10.3390/ani15050676 (PMC11899011; doi:10.3390/ani15050676)

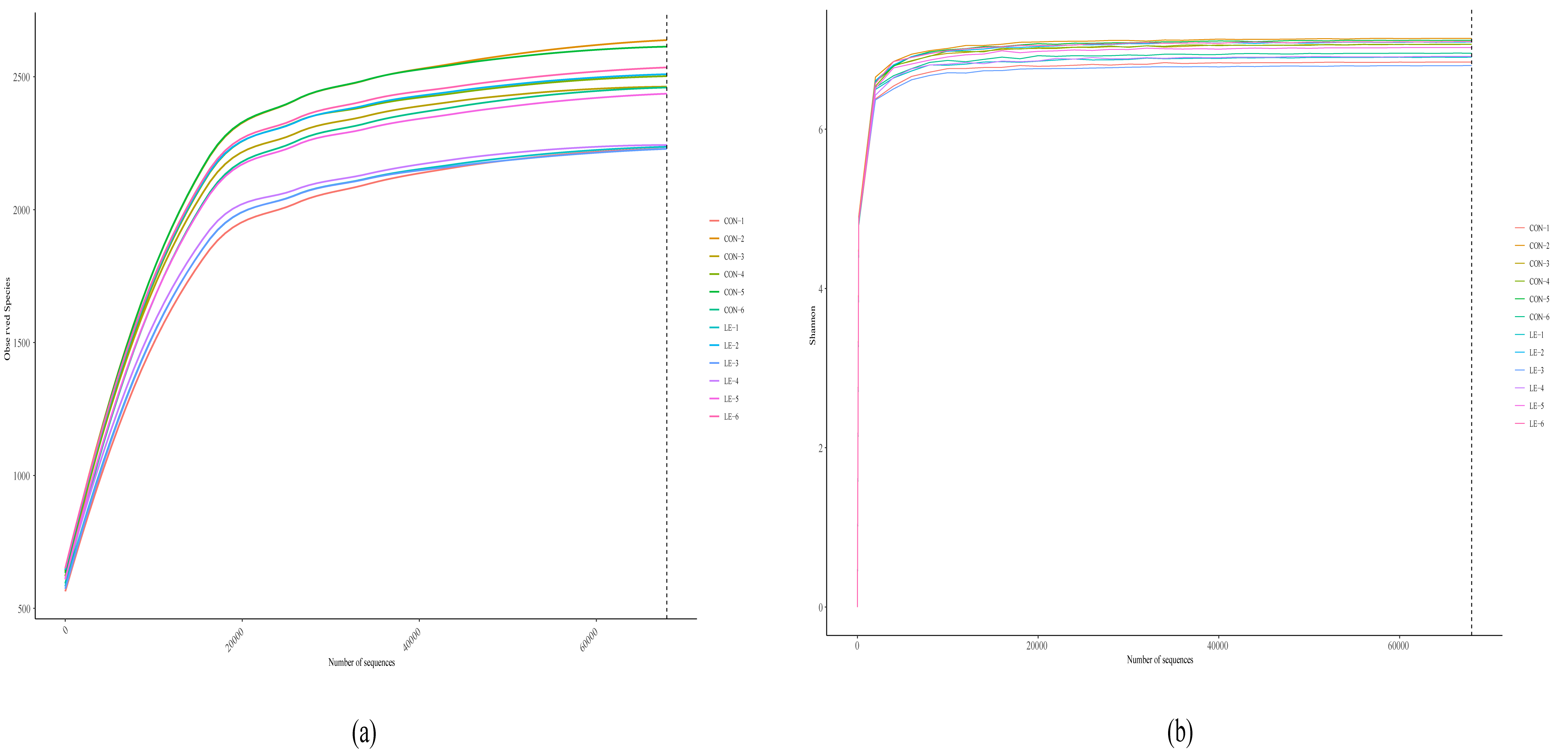

Supplement: Supplementary file 1 [file animals-15-00676-s001.zip › Supplementary materials/Figure S1.tif]
